# Cross-sectional survey on the use and impact of the Danish national antibiotic use guidelines for companion animal practice

**DOI:** 10.1186/s13028-017-0350-8

**Published:** 2017-12-11

**Authors:** Lisbeth Rem Jessen, Tina Møller Sørensen, Zenia Littau Lilja, Maja Kristensen, Tine Hald, Peter Damborg

**Affiliations:** 1 Department of Veterinary Clinical Sciences, University of Copenhagen, Dyrlægevej 16, 1870 Frederiksberg C, Denmark; 2 National Food Institute, Technical University of Denmark, Kemitorvet, Building 204, 2800 Kgs. Lyngby, Denmark; 3 Department of Veterinary and Animal Sciences, University of Copenhagen, Stigbøjlen 4, 1870 Frederiksberg C, Denmark

**Keywords:** Bacteriological testing, Culture, Rational antimicrobial use, Prescription patterns, Pyoderma, Questionnaire, Sensitivity, Susceptibility, Urinary tract infections, UTI

## Abstract

**Background:**

The Danish antibiotic use guidelines for companion animal practice were published by the Danish Veterinary Association in 2012. Since then, national surveillance data indicate a 10% reduction in the total use of antibiotics for companion animals, particularly a marked reduction in the use of third generation cephalosporins. The aim of the study was to assess if and how the guidelines have impacted diagnostic and antibiotic prescription habits of the users, and to identify user perceived barriers to implementation.

**Results:**

An online questionnaire was sent to all 882 members of the Danish Small Animal Veterinary Association in October 2015. The survey was completed by 151 veterinarians. Respondents most frequently consulted the recommendations on skin and urinary tract infections (UTI), and users generally reported a high degree of adherence to the recommendations. Sixty-five per cent indicated that the guidelines had influenced their habits in one or more of the areas being investigated, i.e. perioperative use of antibiotics, use of first line antibiotics for the treatment of pyoderma or UTI, and/or use of microbiological diagnostics. Perioperative use of antibiotics for clean surgeries was uncommon, irrespective of whether respondents had consulted the relevant recommendations or not. On the contrary, significant differences in the prescribing habits between guideline users and non-users were observed for pyoderma and UTI, suggesting an impact of the guidelines towards more prudent antimicrobial use. The diagnostic habits were examined in a subgroup of 63 guideline users. Of those, 19 and 39% reported frequent use of culture and susceptibility (C&S) testing prior to treating pyoderma and UTI respectively, whereas 68–84% reported C&S testing in the event of poor response to treatment or recurrence of infections. The main barriers for implementation of therapeutic recommendations were confidence in old prescribing practices and unavailability of recommended drugs. The main barriers for C&S testing were good experience with empiric treatment, and the owners’ financial situation.

**Conclusions:**

The findings suggest a positive influence of the national antibiotic guidelines on prescription patterns among companion animal practitioners in Denmark. Sustained campaign activity is encouraged and should include promotion of bacteriological testing.

**Electronic supplementary material:**

The online version of this article (10.1186/s13028-017-0350-8) contains supplementary material, which is available to authorized users.

## Background

Antibiotic consumption is widely recognized as the main driver for the selection of antibiotic resistant bacteria, and rational antimicrobial use is therefore considered one of the most important strategies in combatting antimicrobial resistance [1]. Rational antimicrobial use may be defined as usage resulting in clinical resolution while selecting minimally for antimicrobial resistance. Several international and national guidelines for antimicrobial use have been developed over the last decade to help companion animal veterinarians towards this goal [2–6]. Such guidelines are written as clinical practice guidelines indicating diagnostic criteria for—and recommended therapy (drug, dose, duration) of—the most common bacterial infectious diseases in companion animals.

When implemented at hospital or practice level, clinical practice guidelines hold a huge potential to impact prescribing habits of veterinary clinicians and promote prudent use of antibiotics [7].

In Sweden, national antibiotic use guidelines for companion animals [5] were first published in 2003 and later revised in 2009. Since then the use of antibiotics, in particular cephalosporins, fluoroquinolones and potentiated aminopenicillins, has dropped markedly [8]. In a survey conducted in 2012 on antibiotic use and prescribing habits among veterinary practitioners from seven European countries, Swedish practitioners reported a very low use of critically important antibiotics for companion animals [9] and, as the sole country, indicated antibiotic guidelines as the most important factor influencing their prescription habits [10].

In Denmark, national antibiotic use guidelines for companion animals were published in November 2012 [6] and distributed in a booklet format to all members of the Danish Small Animal Association (DSAVA). The guidelines are available also as PDF downloads (Danish and English) and as a smartphone/tablet application. All versions are free of charge. Between 2012 and 2016, national consumption data indicate a 10% reduction in the total use of antibiotics for companion animals, including an almost 40% reduction in the use of third generation cephalosporins [11]. However, it is unknown to what extent such changes may be attributable to the use of the guidelines.

The aim of this survey was to assess if and how the Danish national antibiotic use guidelines for companion animals have impacted diagnostic habits and antibiotic prescription patterns of users, and to identify user perceived barriers to implementation of recommendations.

## Methods

A questionnaire (Additional file 1) was designed to investigate (1) if and to which extent the guidelines were consulted, (2) impact of the guidelines on prescriber habits within the following areas: perioperative use of antibiotics, prescription patterns for infections of the skin and urinary tract (UTI), and use of culture and susceptibility testing (C&S) and, (3) the practitioners attitude to the guidelines, and their perception of the utility and applicability in practice.

The questionnaire was tested in a small focus group of four practitioners before being launched as an electronic survey using TricTrac Student, (http://www.trictrac.com). The survey was accessible from October to November 2015, and during this period a link to the questionnaire was sent twice by e-mail to all 882 members of DSAVA. Participation in the survey was anonymous, and respondents could choose to participate in a draw for prizes (Additional file 2).

The Danish national guidelines consists of different chapters each providing infection-specific diagnostic and therapeutic recommendations. Respondents having consulted a specific set of recommendations (e.g. treatment of skin infections) were defined as “users” of those recommendations, regardless of whether they indicated adherence or not to the recommendations. Respondents who had not consulted the specific set of recommendations were defined as “non-users”.

The questionnaire consisted of 86 questions, of which 73 where multiple choice and 13 were open. Open questions reported in this paper all relate to personal therapeutic habits. The majority of questions were open to all participants. However, questions regarding adherence to specific recommendations were only asked to *users*.

The questionnaire was divided in three parts. In the first part, participants were asked information on gender, age, graduation year, practice location, type of practice, and presence of any written antibiotic policy in their practice. In the second part, participants were asked questions related to their use of—and adherence to—the different recommendations of the guidelines. This included questions related to (i) their perioperative use of antibiotics and choice of treatment for pyoderma and UTI, and (ii) their diagnostic habits with regards to use of C&S. Finally, in the third part, participants were asked questions related to their perception of the guidelines and barriers for implementation. The questionnaire in original language (Danish) is available upon request. The criteria for evaluating adherence are listed in Table 1.

**Table 1:** Criteria for evaluating if specific therapeutic practices were in accordance with the national guideline recommendations

Indication	Assessment of accordance	
	In accordance	Not in accordance
Use of perioperative antibiotics	In 0–10% of clean surgeries	In > 10% of clean surgeries
Treatment of superficial pyoderma	Topical treatment alone Topical treatment in combination with a lincosamide	Topical treatment in combination with systemic antibiotics other than lincosamide Systemic treatment alone
Treatment of cystitis	Amoxicillin or potentiated sulfonamides	Systemic treatment other than amoxicillin or potentiated sulfonamides

Descriptive statistics were performed using Excel 2013, Microsoft Office. Chi square tests were performed for comparison of habits between guideline users and non-users (SAS Enterprise Guide 2013). Study power and margin of error was calculated using EpiInfo v7.2.0.1. P < 0.05 was considered statistically significant.

## Results

The questionnaire was completed by 151 companion animal practitioners, corresponding to 17% (151/882) of the DSAVA members. This resulted in a statistical power of approximately 85%, a confidence level of 95%, and a margin of error of 7.3% for the survey.

**Fig. 1:**
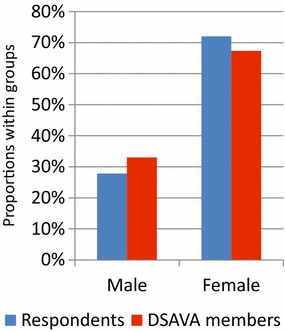
Gender distribution of the 151 respondents and the 882 Danish Small Animal Association (DSAVA) members

**Fig. 2:**
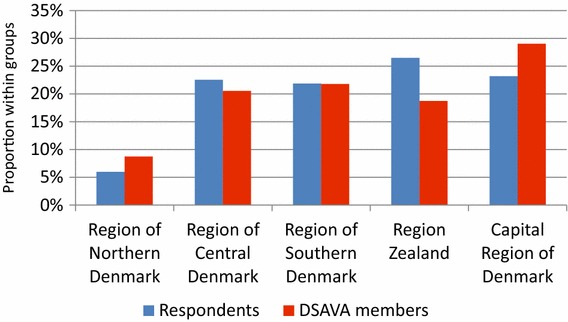
Geographical location in Denmark of the 151 respondents and the 882 Danish Small Animal Association (DSAVA) members

**Fig. 3:**
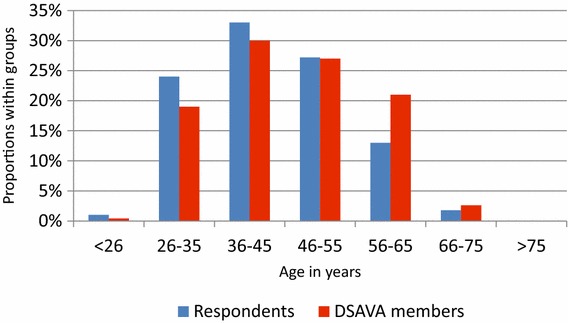
Age distribution of the 151 respondents and the 882 Danish Small Animal Association (DSAVA) members

The distribution of age, gender and geographical location (Additional file 2) of the 151 respondents was similar to that of the entire group of 882 DSAVA members (Figs. 1, 2 and 3). Thirty per cent (46/151) of the clinics had a written antibiotic policy.

### Consultation of the guidelines

Of the 151 respondents, 145 (96%) reported they had consulted the guidelines. Twelve per cent (18/151) had never heard of the smartphone/tablet application, and among those that knew of the application, 59% (78/133) preferred the booklet or PDF format, whereas 38% (51/133) preferred the smartphone/tablet application. Three per cent (4/133) had never used the guidelines or did not know which version they had used.

**Fig. 4:**
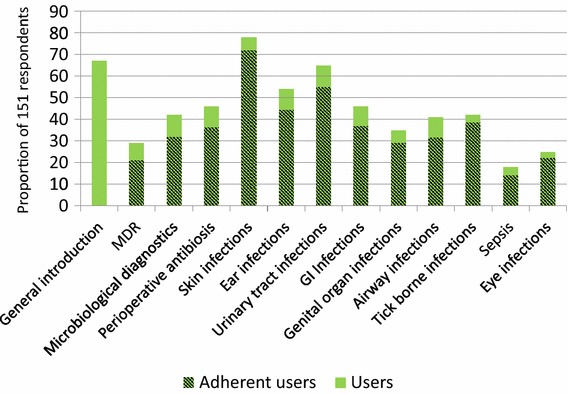
The proportion of respondents consulting the different recommendations of the antibiotic use guidelines (users). Shaded area of the columns are the proportion of users answering yes to the following question: “Do you predominately adhere to the recommendations in this area?”

Respondents most frequently consulted the recommendations on skin infections (78%, 118/151) and UTI (64%, 96/151). Between 73 and 92% (32/44 and 109/118) of users indicated adherence to the recommendations they had consulted (Fig. 4).

### Prescribing habits and diagnostics

In total, 98 out of 151 respondents (65%) answered that the antibiotic guidelines had influenced their habits in one or more of the following areas: use of perioperative antibiotics, choice of treatment for pyoderma and/or UTI, and/or use of culture and susceptibility testing.

### Perioperative antibiotics

In total, 66 out of 151 respondents (44%) indicated that the guidelines had led them to reduce their perioperative use of antibiotics. When asked specifically about their habits, 75% (113/151) reported peri- or postoperative use of antibiotics in less than 10% of clean surgeries, which was in accordance with the recommendations (Table 1). Among the 70 respondents who had consulted the guideline recommendations on perioperative antibiotics (users), this proportion was 81% (57/70), and among the 81 who had not consulted the recommendations (non-users), the proportion was 69% (56/81). There was no significant difference between users and non-users (P = 0.083).

### Skin infections

**Fig. 5:**
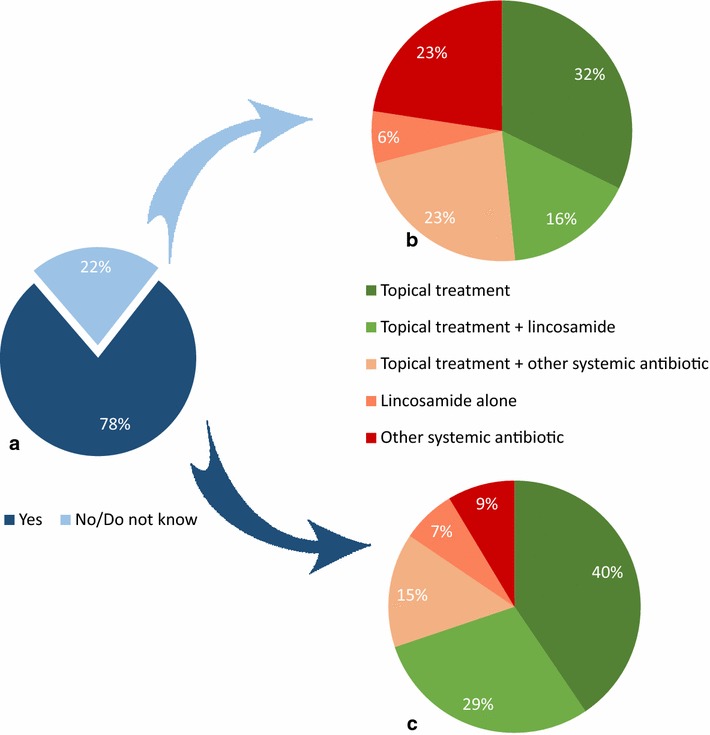
Users and non-users choice of first line treatment for superficial pyoderma. **a** Proportion of the 151 respondents answering yes (users) or no (non-users) to the following question: “Have you consulted the recommendations on skin infections?”. **b** Choice of first line treatment for superficial pyoderma among the 31 non-users. **c** Choice of first line treatment for superficial pyoderma among the 116 users

In total, 68 out of 151 respondents (45%) indicated that the guidelines had influenced their choice of treatment for canine superficial pyoderma. When asked about their first line treatment for canine superficial pyoderma, 65% (96 out of 147 respondents replying to the question) reported use of topical treatment alone or in combination with systemic lincosamides. Both of these treatment regimens are in accordance with the recommendations (Table 1). Among the 116 respondents who had consulted the recommendations on skin infection (users), this proportion was 70% (81/116), and among the 31 who had not consulted the recommendations (non-users) the proportion was 48% (15/31) (Fig. 5). The difference between users and non-users was statistically significant (P = 0.026).

### Urinary tract infections

**Fig. 6:**
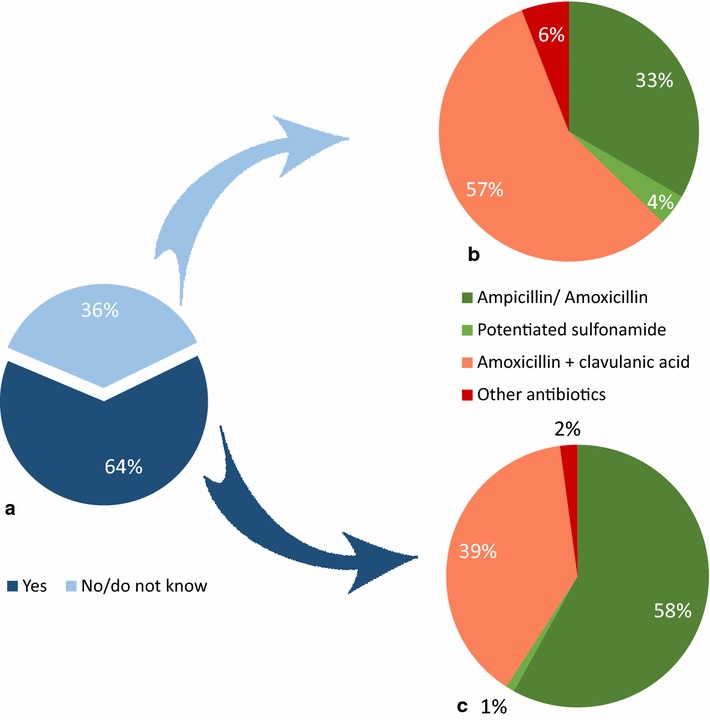
Users and non-users choice of first line treatment for cystitis. **a** Proportion of the 151 respondents answering yes (users) or no (non-users) to the following question: “Have you consulted the recommendations on urinary tract infections?”. **b** Choice of first line treatment for cystitis among the 51 non-users. **c** Choice of first line treatment for cystitis among the 93 users

In total, 36 out of 151 respondents (24%) indicated that the guidelines had influenced their choice of treatment for UTI. When asked about their first line treatment for cystitis, 51% (74 out of 144 respondents replying to the question) reported use of amoxicillin or potentiated sulfonamides in accordance with the recommendations (Table 1). Among the 93 respondents who had consulted the recommendations on UTI (users) this proportion was 59% (55/93), and among the 51 who had not consulted the recommendations (non-users) the proportion was 37% (19/51) (Fig. 6). The difference between users and non-users was statistically significant (P = 0.012).

### Culture and susceptibility testing

Questions regarding C&S testing were intended for all respondents, but by programming error only the 63 respondents who had consulted the recommendations on microbiological diagnostics (users) were asked. In total, 35 of those 63 users (56%) replied that consulting the guidelines had increased their use of C&S testing. The proportions of users performing C&S testing in different suggested clinical scenarios are listed in Table 2.

**Table 2:** Proportion of users performing culture and susceptibility testing in different clinical scenarios

Scenarios	Number of users (%) n = 63
When I treat pyoderma (any type) with systemic antibiotics	12 (19)
When I treat UTI (any type) with antibiotics	25 (39)
When I treat recurrent pyoderma with systemic antibiotics	53 (84)
When I treat recurrent UTI with antibiotics	50 (79)
When I treat rare infections	35 (55)
When empiric treatment fails	43 (68)
I rarely perform C&S	1 (2)
Other	6 (10)

Users of the chapter on microbiological diagnostics answered to the question “In which of the following scenarios do you usually perform C&S?”. Multiple answers were allowed.

### User perception of barriers for implementation

The majority of respondents (95%, 144/151) fully agreed that the national Danish antibiotic use guidelines for companion animals is an important initiative, and 77% (116/151) fully agreed that guidelines can reduce the occurrence of antibiotic resistance in companion animals in Denmark.

The following main barriers for not adhering to the therapeutic recommendations of the guidelines were identified among the respondents: confidence in old prescribing practices (46%, 69/151), unavailability of products registered for dogs and cats (34%, 52/151), difficulties dosing the drug (e.g. due to odd tablet size) (31%, 47/151), costs (30%, 46/151), lack of time for consulting the guidelines (25%, 38/151), limited number of antibiotic drugs available on site (23%, 35/151), owners difficulties in administering drugs (18%, 27/151).

The main barriers for performing C&S among the 63 users were the owners’ financial situation (70%, 44/63) and good experience with empiric treatment (60%, 38/63). Furthermore, 25% (16/63) of users noted that it is not necessary to perform C&S as often as the guideline recommends, and 21% (13/63) noted that they never or rarely encounter infections with antibiotic resistant bacteria.

## Discussion

The survey indicates that the guidelines have been positively received among the respondents, of which the majority indicated adherence to the recommendations. To better investigate the direct and indirect impact of guidelines on prescription habits, a comparison of actual antibiotic prescriptions before and after guideline publication would be ideal. Since such data were not available, we compared the choice of treatment in specific scenarios for users vs. non-users of the guidelines. This comparison showed a significant difference between the two groups with regard to treatment of pyoderma and UTI (Figs. 5 and 6). Although the causal relationship cannot be established with certainty, the results suggest that the guidelines have influenced the habits of its readers towards more rational antimicrobial use. On the other hand, there was no apparent effect of the guidelines on perioperative use of antibiotics, since both users and non-users reported very limited use of antibiotics for this indication. This result likely reflects the restrictive use of antibiotics in surgery that is well established amongst veterinarians in Denmark, which could be due to many years of teaching aseptic surgical techniques to veterinary students. Accordingly, the guideline emphasizes what is generally known about perioperative antibiotic use, instead of changing a treatment paradigm.

When comparing the results for pyoderma and UTI, the survey shows that recommendations for skin infections were not only being consulted more frequently, they are also being converted into practice more successfully. Even among the practitioners not consulting the guidelines on pyoderma (non-users) their choices were nearly 50% in accordance with the recommendations. This result is probably explained by campaigning activity conducted by the Danish Veterinary Dermatological Network (DVEN) prior to and following the publication of the guidelines, and illustrates the great importance of active dissemination and campaigns.

The Danish guidelines recommend frequent use of C&S testing, including for all cases of suspected pyoderma and UTI. One rationale behind this recommendation is the relatively high consumption of antibiotics for these common indications [9, 12]. Furthermore, multidrug-resistant pathogens like methicillin-resistant *Staphylococcus pseudintermedius* (MRSP) and extended-spectrum beta-lactamase (ESBL)-producing *Enterobacteriaceae* have emerged in companion animals, and these pathogens occur primarily in the skin and the urinary tract, respectively [13]. Unfortunately, the habits for C&S testing were only examined among the respondents consulting the section on microbiological diagnostics, and the difference between guideline users and non-users could not be assessed. However, the survey clearly indicates that, even though 56% of users reported an increased use of C&S testing this is not routinely performed for pyoderma and UTI, except in recurrent cases (Table 2). The result is not surprising, as recommending broad use of C&S testing represents a major shift of paradigm, which takes time to implement. In a European survey from 2012 [10], 51% of companion animal practitioners reported using C&S testing for complicated or non-responding cases only, and 16% reported to rarely or never use it. Interestingly, broad use of sensitivity testing was much more widespread among Swedish practitioners participating in that survey.

As anticipated from other studies of veterinary antibiotic prescription patterns [10, 14], financial costs were a commonly reported barrier in our study, and 70% of respondents reported the owners financial situation to be a limiting factor for performing bacteriological diagnostics. This is in line with surveys on veterinary prescription patterns in North America where 65% [14] and 84% [15] of participants reported costs as a barrier to recommending C&S testing. In that regard it should be noted that the costs of C&S testing are relatively modest, at least in Scandinavia, and in most instances lower than the costs associated with treatment failure (e.g. repeated veterinary consultations and antibiotic treatments). Regardless, as long as diagnostic testing relies on owner/client economy, test costs will remain a potential barrier to antibiotic stewardship. In light of this situation, companion animal veterinarians must educate owners on the importance and benefits of this practice prior to antimicrobial treatment.

The survey indicates that a variety of factors influence the adherence to therapeutic recommendations, and that practical barriers such as unavailability of recommended drugs for veterinary use, odd tablet size, and ease of administration play an important role. Similarly, ease of administration was identified as a factor influencing antibiotic prescription patterns in a cross sectional survey among companion animal veterinarians in the UK [16]. To exemplify the problems of availability, potentiated sulfonamides are recommended as first choice for several conditions in the guidelines, but unfortunately from March 2015 no oral product for companion animal use has been available on the Danish market. Other commonly recommended drugs, including amoxicillin have been in short supply on the Danish market periodically. Such barriers are difficult to foresee and to solve by the authors of the guidelines, who base recommendations on clinical effect and knowledge of selection pressure and critical importance of drugs. Recommending more than one drug for each condition may partly overcome this problem.

The response rate on this survey was 17%, which is low in a small and well defined population with a presumed interest in the topic. The reason for this is unknown but falls within a general trend of decreased response rates in epidemiological studies [17, 18]. The response rate might have improved by directly contacting the practitioners, or offering further incentives to participants. Irrespectively, a low response rate is not necessarily problematic if demographic representation and study power is adequate [19], which was the case in our study. Of importance, the investigation relied on voluntary participation. This could have biased the results (selection bias) towards a higher degree of adherence to the guidelines, as the respondents may have an increased interest in antibiotic use.

The criteria for evaluating if specific therapeutic practices were in accordance with the guideline recommendations were to some extent subjective (Table 1). To exemplify, we considered topical antiseptic treatment both with and without a lincosamide as appropriate for treatment of superficial pyoderma, even though the guidelines recommend topical treatment alone as the first line. The rationale behind the decision was the lack of distinction between first episode cases and recurring cases in the questionnaire. Many respondents therefore added comments that topical treatment was their first choice for superficial pyoderma with combination therapy being applied only for difficult, non-responsive, or recurrent cases.

## Conclusions

The findings suggest a positive influence of the national antibiotic use guidelines on prescription pattern among companion animal practitioners in Denmark. This is supported by national consumption data, since the use of particularly some important broad-spectrum drugs like third generation cephalosporins has markedly dropped since the guidelines were published. The vast majority of respondents had consulted the guidelines and perceived them as useful despite issues such as limited availability of certain drugs. Future campaigns are strongly encouraged to promote implementation of the recommendations in practice. Campaigns should focus on infections for which antibiotics are commonly prescribed, and on the use of diagnostic testing.

## Additional files

**Additional file 1.** Questionnaire (English translation).

**Additional file 2.** Map of the five Danish regions.
