# Supplementary material for: Cross-sectional survey on the use and impact of the Danish national antibiotic use guidelines for companion animal practice
Source: Acta Vet Scand. 2017 Dec 11;59:81. doi: 10.1186/s13028-017-0350-8 (PMC5725655; doi:10.1186/s13028-017-0350-8)
Supplement: Supplementary file 1 — Additional file 1. Questionnaire (English translation). [file 13028_2017_350_MOESM1_ESM.pdf]

Cross-sectional survey on the use and impact of the Danish national antibiotic use guidelines for companion animal practice

Questionnaire – English translation

Personal information/Clinic related information:

1) Gender
   - o Male/Female
2) Age
   a. Textbox
3) In which year did you graduate?
   a. Textbox
4) How many years have you worked in a small animal practice?
   a. Textbox
5) In the clinic where I work, I am:
   a. Owner/co-owner
   b. Employed
6) In which region do you work?
   a. Northern Jutland
   b. Middle Jutland
   c. Southern Denmark
   d. Zealand
   e. Capital
7) Which type of practice do you work in?
   a. Mixed
   b. Small animals exclusively
8) How many veterinarians are associated with the practice in which you work?
   a. Textbox
9) Is the clinic ISO certified?
   a. Yes/No

General questions regarding the antibiotic use guidelines for companion animal practice:

10) Does the practice you work in have a written antibiotic policy?
    a) Yes/No

11) Are the recommendations from the antibiotic handbook fully or partially used in this policy? (only if yes to question 10)
    a) Fully
    b) Partially
    c) No

12) From where did you hear about the antibiotic use guidelines for companion animal practice? (multiple answers allowed)
    a) From courses
    b) Via my education
    c) It was sent to me
    d) DVT/Journalen
    e) DDD's newsletter
    f) SvHKS' website
    g) Non-veterinary medias (newspaper/radio/tv)
    h) From colleagues
    i) I have not heard about it

13) Are you aware that the antibiotic use guidelines for companion animal practice are available as an App?
    a) Yes/No

14) Which version of the antibiotic use guidelines for companion animal practice do you use most often? <u>(only if yes to question 13)</u>
    a) Booklet/PDF
    b) App
    c) I do not know
    d) I do not use the antibiotic use guidelines for companion animal practice

15) In which situations will you likely reference the antibiotic use guidelines for companion animal practice?
    a) Before empirical antibiotic treatment
    b) When my empirical antibiotic treatment fails
    c) In relation to operations
    d) When I have to interpret a susceptibility test
    e) I relation to infections I do not often see
    f) When I need instructions for sampling
    g) I do not use it
    h) Other (explain)

Questions regarding the individual chapters in the antibiotic use guidelines for companion animal practice:

16) Have you actively sought information in one or more of the following 3 chapters: *General principles regarding rational use of antibiotics (chapter 1), Antibiotic use in family animals (chapter 2), and Handling of antibiotics and other medicines (chapter 7)?*
    a) Yes
    b) No
    c) I do not know/do not remember

17) Do you have comments to one or more of the above-mentioned chapters?
    a) Yes (textbox)
    b) No

18) Have you actively sought information in the chapter *New multidrug resistant bacteria in family pets?*
    a) Yes
    b) No
    c) I do not know/do not remember

19) Have you applied the therapeutic recommendations from the chapter *New multidrug resistant bacteria in family animals?* <u>(only if yes to question 18)</u>
    a) Predominantly yes
    b) Predominantly no
    c) I do not know/do not remember

20) Why have you chosen to apply the therapeutic recommendations from the chapter *New multidrug resistant bacteria in family pets?* (multiple answers allowed) <u>(only if predominantly yes to question 19)</u>
    a) In this chapter, the recommendations agree with what is possible for me to comply with in practice
    b) This chapter provides good guidelines for therapy of multidrug resistant infections
    c) I think it is important to treat multidrug resistant infections correctly
    d) I rarely see these types of infections, therefore I do not have my own protocol
    e) Other

21) Why have you chosen **not** to apply the therapeutic recommendations from the chapter *New multidrug resistant bacteria in family pets?* (multiple answers allowed) <u>(only if predominantly no to question 19)</u>
    a) The recommendations in this chapter are not possible for me to comply with in practice
    b) I have found conflicting information about the choice of antibiotics other places
    c) I have good experience with my own protocol for treatment of these kinds of infections
    d) I have exclusively sought information in the chapter out of interest
    e) I am subject to a therapeutic protocol at my workplace
    f) I have not had the time to familiarise myself with the recommendations

g) Other (textbox)

22) Have you actively sought information in the chapter *Recommendations regarding microbiological diagnostics?*
    a) Yes
    b) No
    c) I do not know/do not remember

23) Have you applied the recommendations from the chapter *Recommendations regarding microbiological diagnostics?* <u>(only if yes to question 22)</u>
    a) Predominantly yes
    b) Predominantly no
    c) I do not know/do not remember

24) Why have you chosen to apply the recommendations from the chapter *Recommendations regarding microbiological diagnostics?* (multiple answers allowed) <u>(only if predominantly yes to question 23)</u>
    a) In this chapter, the recommendations agree with what is possible for me to comply with in practice
    b) This chapter provides good guidelines for sampling in order to culture
    c) This chapter provides a good explanation on how to interpret a susceptibility test result
    d) Other

25) Why have you chosen **not** to apply the recommendations from the chapter *Recommendations regarding microbiological diagnostics?* (multiple answers allowed) <u>(only if predominantly no to question 23)</u>
    a) The recommendations in this chapter are not possible for me to comply with in practice
    b) I most often have a good response on my empirical treatment and therefore I rarely culture
    c) The owner's economic situation is often a limiting factor and therefore I rarely culture
    d) I have found conflicting information about microbiological diagnostics other places
    e) I have exclusively sought information in the chapter out of interest
    f) I am subject to other guidelines for diagnostics at my workplace
    g) I have not had the time to familiarise myself with the recommendations
    h) Other (textbox)

26) Which situations will prompt you to carry out culture and susceptibility testing? (multiple answers allowed) <u>(only if yes to question 22 – error in the electronic survey)</u>
    a) When I want to treat systemically with antibiotics against skin infections (irrespective of type)
    b) When I want to treat systemically with antibiotics against urinary tract infections (irrespective of type)
    c) When there are recurrent infections in the skin
    d) When there are recurrent infections in the urinary tract
    e) When there are infections I rarely see
    f) In general, when my empirical treatment is ineffective
    g) I rarely/never culture
    h) Other (textbox)

27) How often do you perform culture and susceptibility testing today, compared to before the antibiotic use guidelines for companion animal practice was published (November 2012)? <u>(only if yes to question 22 – error in the electronic survey)</u>
    a) Less
    b) Unchanged
    c) More
    d) I do not know/do not remember
    e) Not relevant, as I started working within a practice after November 2012 (when guidelines were published)

28) In the cases where you do not apply the recommendations from the antibiotic use guidelines for companion animal practice in relation to culture, it is due to: (multiple answers allowed) <u>(only if yes to question 22 – error in the electronic survey)</u>

a) I have not received sufficient instruction on how to use the antibiotic use guidelines for companion animal practice
b) During a busy weekday, it takes too long to reference the antibiotic use guidelines for companion animal practice
c) I never/rarely experience resistant bacteria in the clinic
d) I do not believe it is necessary to culture as often as the antibiotic use guidelines for companion animal practice recommends
e) I often see a good effect with my empirical treatment
f) The owner's economic situation is often a limitation in terms of complying with the recommendations
g) I have not had the time to familiarise myself with the recommendations
h) I have found conflicting information regarding culture other places
i) Other (textbox)

29) Have you actively sought information in the chapter *Perioperative use of antibiotics?*
a) Yes
b) No
c) I do not know/do not remember

30) Have you applied the recommendations from the chapter *Perioperative use of antibiotics?* <u>(only if yes to question 29)</u>
a) Predominantly yes
b) Predominantly no
c) I do not know/do not remember

31) Why have you chosen to apply the recommendations from the chapter *Perioperative use of antibiotics?* (multiple answers allowed) <u>(only if predominantly yes to question 30)</u>
a) In this chapter, the recommendations agree with what is possible for me to comply with in practice
b) This chapter provides me with good guidelines to evaluate for which patients it is necessary to apply perioperative antibiosis
c) This chapter provides me with good guidelines to prevent and treat postoperative infections
d) I often have problems with postoperative infections
e) Other

32) Why have you chosen **not** to apply the recommendations from the chapter *Perioperative use of antibiotics?* (multiple answers allowed) <u>(only if predominantly no to question 30)</u>
a) I have good experience with my own protocol for perioperative antibiosis
b) It is difficult to apply the recommendations in the chapter in practice
c) I often have problems with postoperative infections
d) I have exclusively sought information in the chapter out of interest
e) I am subject to a perioperative protocol at my workplace
f) I have found conflicting information about perioperative antibiosis other places
g) Other (textbox)

33) How often do you apply postoperative antibiosis in relation to clean operations (e.g. castration, ovariectomy, ovariohysterectomy, explorative laparotomy)?
a) More than 90% of the operations
b) 51-90% of the operations
c) 11-50% of the operations
d) 1-10% of the operations
e) 0% of the operations

34) For which reason do you often apply postoperative antibiosis for this type of operation? <u>(only if a or b to question 33)</u> (multiple answers allowed)
a) I often experience a break in asepsis
b) The operations are often long-lasting (more than 90 minutes)
c) I often experience postoperative infections
d) It is the procedure at my workplace
e) Other (textbox)

35) Has it influenced your habits in relation to perioperative antibiosis that the antibiotic use guidelines for companion animal practice was published (November 2012)?
    a) Yes, I less frequently use perioperative antibiosis
    b) Yes, I more frequently use perioperative antibiosis
    c) No, my habits are unchanged
    d) Not relevant, as I started working within a practice after November 2012

36) Have you actively sought information in the chapter *Skin?*
    a) Yes
    b) No
    c) I do not know/do not remember

37) Have you applied the therapeutic recommendations from the chapter *Skin?* <u>(only if yes to question 36)</u>
    a) Predominantly yes
    b) Predominantly no
    c) I do not know/do not remember

38) Why have you chosen to apply the therapeutic recommendations from the chapter *Skin?* (multiple answers allowed) <u>(only if predominantly yes to question 37)</u>
    a) In this chapter, the recommendations agree with what is possible for me to comply with in practice
    b) This chapter provides good therapeutic guidelines
    c) My knowledge about antimicrobial therapy of infections in the skin was limited before the antibiotic handbook was published (before November 2012)
    d) I frequently have patients with infections in the skin and therefore find the recommendations relevant
    e) I rarely have patients with infections in the skin and therefore I need instructions when I attend this type of patient
    f) Other

39) Why have you chosen **not** to apply the therapeutic recommendations from the chapter *Skin?* (multiple answers allowed) <u>(only if predominantly no to question 37)</u>
    a) I have good experience with my own protocol for patients with skin infections
    b) It is difficult to apply the recommended therapy in practice
    c) The owner's economic situation is often a limitation in terms of complying with the recommendations
    d) I have found conflicting information about therapy of skin infections other places
    e) I have exclusively sought information in the chapter out of interest
    f) I am subject to a therapeutic protocol at my workplace
    g) I have not had the time to familiarise myself with the recommendations
    h) Other (textbox)

40) Which therapy is your empirical first choice for superficial dermatitis, including antiseptic agents/systemic antibiosis? (ingredient or product name):
    a) Textbox

41) Has it influenced your empirical first choice for superficial dermatitis that the antibiotic handbook was published (November 2012)?
    a) Yes
    b) No
    c) I do not know
    d) Not relevant, as I started working within a practice after November 2012

42) What was your empirical first choice for superficial dermatitis before the antibiotic handbook was published (before November 2012)? <u>(only if yes to question 41)</u>
    a) Textbox
    b) I do not know/do not remember

43) Have you actively sought information in the chapter *Ears?*
    a) Yes

b) No
c) I do not know/do not remember

44) Have you applied the therapeutic recommendations from the chapter *Ears?* (only if yes to question 43)
a) Predominantly yes
b) Predominantly no
c) I do not know/do not remember

45) Why have you chosen to apply the therapeutic recommendations from the chapter *Ears?* (multiple answers allowed) (only if predominantly yes to question 44)
a) In this chapter, the recommendations agree with what is possible for me to comply with in practice
b) This chapter provides good therapeutic guidelines
c) My knowledge about antimicrobial therapy of infections in the ears was limited before the antibiotic handbook was published (before November 2012)
d) I frequently have patients with ear infections and therefore find the recommendations relevant
e) I rarely have patients with ear infections, therefore I need instructions when I attend this type of patient
f) Other

46) Why have you chosen **not** to apply the therapeutic recommendations from the chapter *Ears?* (multiple answers allowed) (only if predominantly no to question 44)
a) I have good experience with my own protocol for patients with ear infections
b) It is difficult to apply the recommended therapy in practice
c) I have found conflicting information about therapy of ear infections other places
d) I have exclusively sought information in the chapter out of interest
e) I am subject to a therapeutic protocol at my workplace
f) I have not had the time to familiarise myself with the recommendations in this chapter
g) The owner's economic situation is often a limitation in terms of complying with the recommendations
h) Other (textbox)

47) Have you actively sought information in the chapter *Urinary tract?*
a) Yes
b) No
c) I do not know/do not remember

48) Have you applied the therapeutic recommendations from the chapter *Urinary tract?* (only if yes to question 47)
a) Predominantly yes
b) Predominantly no
c) I do not know/do not remember

49) Why have you chosen to apply the therapeutic recommendations from the chapter *Urinary tract?* (multiple answers allowed) (only if predominantly yes to question 48)
a) In this chapter, the recommendations agree with what is possible for me to comply with in practice
b) This chapter provides good therapeutic guidelines
c) My knowledge about antimicrobial therapy of infections in the urinary tract was limited before the antibiotic handbook was published (before November 2012)
d) I frequently have patients with infections in the urinary tract and therefore find the recommendations relevant
e) I rarely have patients with infections in the urinary tract and therefore need instructions when I attend this type of patient
f) Other

50) Why have you chosen **not** to apply the therapeutic recommendations from the chapter *Urinary tract?* (multiple answers allowed) (only if predominantly no to question 48)
a) I have good experience with my own protocol for patients with urinary tract infections
b) It is difficult to apply the recommended therapy in practice
c) The owner's economic situation is often a limitation in terms of complying with the recommendations

d) I have found conflicting information about therapy of urinary tract infections other places
e) I have exclusively sought information in the chapter out of interest
f) I am subject to a therapeutic protocol at my workplace
g) I have not had the time to familiarise myself with the recommendations
h) Other (textbox)

51) Which antibiotic is your empirical first choice for infections in the lower urinary tract/cystitis? (please state ingredient or product name):
a) Textbox

52) Has it influenced your empirical first choice for lower urinary tract infections/cystitis that the antibiotic use guidelines for companion animal practice were published (November 2012)?
a) Yes
b) No
c) I do not know
d) Not relevant, as I started working within a practice after November 2012

53) What was your empirical first choice for lower urinary tract infections/cystitis before the antibiotic use guidelines for companion animal practice was published? <u>(only if yes to question 52)</u>
a) Textbox
b) I do not know

54) Have you actively sought information in the chapter *Oral cavity and gastrointestinal tract?*
a) Yes
b) No
c) I do not know/do not remember

55) Have you applied the therapeutic recommendations from the chapter *Oral cavity and gastrointestinal tract?* <u>(only if yes to question 54)</u>
a) Predominantly yes
b) Predominantly no
c) I do not know/do not remember

56) Why have you chosen to apply the therapeutic recommendations from the chapter *Oral cavity and gastrointestinal tract?* (multiple answers allowed) <u>(only if predominantly yes to question 55)</u>
a) In this chapter, the recommendations agree with what is possible for me to comply with in practice
b) This chapter provides good therapeutic guidelines
c) My knowledge about antimicrobial therapy of infections in the oral cavity and gastrointestinal tract was limited before the antibiotic handbook was published (before November 2012)
d) I frequently have patients with infections in the oral cavity and gastrointestinal tract and therefore find the recommendations relevant
e) I rarely have patients with infections in the oral cavity and gastrointestinal tract and therefore need instructions when I attend this type of patient
f) Other

57) Why have you chosen **not** to apply the therapeutic recommendations from the chapter *Oral cavity and gastrointestinal tract?* (multiple answers allowed) <u>(only if predominantly no to question 55)</u>
a) I have good experience with my own protocol for patients with infections in the oral cavity and gastrointestinal tract
b) It is difficult to apply the recommended therapy in practice
c) I have found conflicting information about therapy of infections in the oral cavity and gastrointestinal tract other places
d) I have exclusively sought information in the chapter out of interest
e) I am subject to a therapeutic protocol at my workplace
f) I have not had the time to familiarise myself with the recommendations
g) The owner's economic situation is often a limitation in terms of complying with the recommendations

58) Have you actively sought information in the chapter *Genital organs?*

a) Yes
b) No
c) I do not know/do not remember

59) Have you applied the therapeutic recommendations from the chapter *Genital organs?* <u>(only if yes to question 58)</u>
   a) Predominantly yes
   b) Predominantly no
   c) I do not know/do not remember

60) Why have you chosen to apply the therapeutic recommendations from the chapter *Genital organs?* (multiple answers allowed) <u>(only if predominantly yes to question 59)</u>
   a) In this chapter, the recommendations agree with what is possible for me to comply with in practice
   b) This chapter provides good therapeutic guidelines
   c) My knowledge about antimicrobial therapy of infections in the genital organs was limited before the antibiotic handbook was published (before November 2012)
   d) I frequently have patients with infections in genital organs and therefore find the recommendations relevant
   e) I rarely have patients with infections in genital organs and therefore need instructions when I attend this type of patient
   f) Other

61) Why have you chosen **not** to apply the therapeutic recommendations from the chapter *Genital organs?* (multiple answers allowed) <u>(only if predominantly no to question 59)</u>
   a) I have good experience with my own protocol for patients with genital infections
   b) It is difficult to apply the recommended therapy in practice
   c) I have found conflicting information about therapy of infections in the genital organs other places
   d) I have exclusively sought information in the chapter out of interest
   e) I am subject to a therapeutic protocol at my workplace
   f) I have not had the time to familiarise myself with the recommendations
   g) The owner's economic situation is often a limitation in terms of complying with the recommendations
   h) Other (textbox)

62) Have you actively sought information in the chapter *Respiratory tract?*
   a) Yes
   b) No
   c) I do not know/do not remember

63) Have you applied the therapeutic recommendations from the chapter *Respiratory tract?* <u>(only if yes to question 62)</u>
   a) Predominantly yes
   b) Predominantly no
   c) I do not know/do not remember

64) Why have you chosen to apply the therapeutic recommendations from the chapter *Respiratory tract?* (multiple answers allowed) <u>(only if predominantly yes to question 63)</u>
   a) In this chapter, the recommendations agree with what is possible for me to comply with in practice
   b) This chapter provides good therapeutic guidelines
   c) My knowledge about antimicrobial therapy of infections in the respiratory tract was limited before the antibiotic handbook was published (before November 2012)
   d) I frequently have patients with infections in the respiratory tract and therefore find the recommendations relevant
   e) I rarely have patients with infections in the respiratory tract and therefore need instructions when I attend this type of patient
   f) Other

65) Why have you chosen **not** to apply the therapeutic recommendations from the chapter *Respiratory tract?* (multiple answers allowed) <u>(only if predominantly no to question 63)</u>

a) I have good experience with my own protocol for patients with respiratory tract infections
b) It is difficult to apply the recommended therapy in practice
c) I have found conflicting information about therapy of respiratory tract infections other places
d) I have exclusively sought information in the chapter out of interest
e) I am subject to a therapeutic protocol at my workplace
f) I have not had the time to familiarise myself with the recommendations
g) The owner's economic situation is often a limitation in terms of complying with the recommendations
h) Other (textbox)

66) Have you actively sought information in the chapter *Tick-borne infections?*
a) Yes
b) No
c) I do not know/do not remember

67) Have you applied the therapeutic recommendations from the chapter *Tick-borne infections?* (only if yes to question 66)
a) Predominantly yes
b) Predominantly no
c) I do not know/do not remember

68) Why have you chosen to apply the therapeutic recommendations from the chapter *Tick-borne infections?* (multiple answers allowed) (only if predominantly yes to question 67)
a) In this chapter, the recommendations agree with what is possible for me to comply with in practice
b) This chapter provides good therapeutic guidelines
c) My knowledge about therapy of tick-borne infections was limited before the antibiotic handbook was published (before November 2012)
d) I frequently have patients with tick-borne infections and therefore find the recommendations relevant
e) I rarely have patients with tick-borne infections and therefore need instructions when I attend this type of patient
f) Other

69) Why have you chosen **not** to apply the therapeutic recommendations from the chapter *Tick-borne infections?* (multiple answers allowed) (only if predominantly no to question 67)
a) I have good experience with my own protocol for patients with tick-borne infections
b) It is difficult to apply the recommended therapy in practice
c) I have found conflicting information about therapy of tick-borne infections other places
d) I have exclusively sought information in the chapter out of interest
e) I am subject to a therapeutic protocol at my workplace
f) I have not had the time to familiarise myself with the recommendations
g) The owner's economic situation is often a limitation in terms of complying with the recommendations
h) Other (textbox)

70) Have you actively sought information in the chapter *Sepsis?*
a) Yes
b) No
c) I do not know/do not remember

71) Have you applied the therapeutic recommendations from the chapter *Sepsis?* (only if yes to question 70)
a) Predominantly yes
b) Predominantly no
c) I do not know/do not remember

72) Why have you chosen to apply the therapeutic recommendations from the chapter *Sepsis?* (multiple answers allowed) (only if predominantly yes to question 71)
a) In this chapter, the recommendations agree with what is possible for me to comply with in practice
b) This chapter provides good therapeutic guidelines
c) My knowledge about antimicrobial therapy of sepsis was limited before the antibiotic handbook was published (before November 2012)

d) I frequently have patients with sepsis and therefore find the recommendations relevant
e) I rarely have patients with sepsis and therefore need instructions when I attend this type of patient
f) Other

73) Why have you chosen **not** to apply the therapeutic recommendations from the chapter *Sepsis?* (multiple answers allowed) <u>(only if predominantly no to question 71)</u>
a) I have good experience with my own protocol for patients with sepsis
b) It is difficult to apply the recommended therapy in practice
c) I have found conflicting information about therapy of sepsis other places
d) I have exclusively sought information in the chapter out of interest
e) I am subject to a therapeutic protocol at my workplace
f) I have not had the time to familiarise myself with the recommendations
g) The owner's economic situation is often a limitation in terms of complying with the recommendations
h) Other (textbox)

74) Have you actively sought information in the chapter *Eyes?*
a) Yes
b) No
c) I do not know/do not remember

75) Have you applied the therapeutic recommendations from the chapter *Eyes?* <u>(only if yes to question 74)</u>
a) Predominantly yes
b) Predominantly no
c) I do not know/do not remember

76) Why have you chosen to apply the therapeutic recommendations from the chapter *Eyes?* (multiple answers allowed) <u>(only if predominantly yes to question 75)</u>
a) In this chapter, the recommendations agree with what is possible for me to comply with in practice
b) This chapter provides good therapeutic guidelines
c) My knowledge about antimicrobial therapy of infections in the eyes was limited before the antibiotic handbook was published (before November 2012)
d) I frequently have patients with infections in the eyes and therefore find the recommendations relevant
e) I rarely have patients with infections in the eyes and therefore need instructions when I attend this type of patient
f) Other

77) Why have you chosen **not** to apply the therapeutic recommendations from the chapter *Eyes?* (multiple answers allowed) <u>(only if predominantly no to question 75)</u>
a) I have good experience with my own protocol for patients with eye infections
b) It is difficult to apply the recommended therapy in practice
c) I have found conflicting information about therapy of eye infections other places
d) I have exclusively sought information in the chapter out of interest
e) I am subject to a therapeutic protocol at my workplace
f) I have not had the time to familiarise myself with the recommendations
g) The owner's economic situation is often a limitation in terms of complying with the recommendations
h) Other (textbox)

78) If there is one or more chapters you have not sought information in, is there a special reason for this?
a) Textbox
b) I do not know
c) I have sought information in all chapters

How much do you agree with the following statements: The antibiotic use guidelines for companion animal practice…
(5 scales; completely agree, partly agree, neither/nor, partly disagree, completely disagree)
79) Is a good initiative
80) Can help in decreasing the development of resistance
81) Can strengthen my arguments to colleagues when I decide to culture

82) Can strengthen my arguments to colleagues/bosses when I choose not to give antibiotic treatment (e.g. for topical antiseptic treatment)
83) Can strengthen my arguments to colleagues/superiors when I choose a more narrow-spectrum antibiotic, which does not have quite as guaranteed effect as a more broad-spectrum choice
84) Gives me professional satisfaction to follow the latest recommendations
85) Is a good idea but does not work in everyday practice
86) Is unnecessary

Challenges in implementing the antibiotic use guidelines for companion animal practice:

87) Do the recommended antibiotics in antibiotic use guidelines for companion animal practice correspond to what is possible to comply with in practice?
    a) Yes, always
    b) Often
    c) Sometimes
    d) No, rarely

88) What are the two most common reasons for why the recommended antibiotics in the handbook cannot be applied in practice? (multiple answers allowed)
    a) The product is not registered for use with the species concerned
    b) The product is difficult to dose, as it does not exist in suitable strength for the species
    c) The recommended antibiotic often does not exist in a suitable package sizes
    d) The recommended antibiotic is often found in a dosage form, which is difficult for the owner to administer at home
    e) Economic situation
    f) Other
    g) For which products (product name or ingredient) do you have one or more of the above-mentioned problems? (textbox)

89) When you do **not** apply the **therapeutic** recommendations in the antibiotic use guidelines for companion animal practice, it is because: (multiple answers allowed)
    a) The product is not registered for use with the species concerned
    b) The product is difficult to dose, as it does not exist in suitable strength for the species concerned
    c) The recommended antibiotic often does not exist in a suitable package size
    d) The recommended antibiotic is often found in a dosage form, which is difficult for the owner to administer at home
    e) We have a limited selection of antibiotics in stock in our practice
    f) I have not received sufficient instruction on how to use the antibiotic use guidelines for companion animal practice
    g) During a busy weekday, it takes too long to reference the antibiotic use guidelines for companion animal practice
    h) I feel most safe when I use drugs I have experience with
    i) Economic situation
    j) Other (textbox)

90) Apart from the antibiotic use guidelines for companion animal practice, what influences your choice of antibiotics?
    a) Textbox
    b) I do not know

Possible improvements on the antibiotic use guidelines for companion animal practice:

91) Which of the following initiatives would increase your use of the antibiotic use guidelines for companion animal practice? (multiple answers allowed)
    a) More meetings/lectures about the use of the antibiotic use guidelines for companion animal practice
    b) Greater agreement between recommended antibiotics and what is possible to comply with in practice (e.g. package sizes, drug strength, registration with certain species)

c) If my workplace introduced the antibiotic use guidelines for companion animal practice as a general guideline when there is a need for antibiosis
d) A list of the most recommended antibiotics that should be in stock in the clinic
e) If the app became available for Windows phones
f) Other (textbox)
g) I do not know

92) Mention 1-3 good things about the antibiotic use guidelines for companion animal practice as it is now:
a) (textbox)
b) I do not know

93) Mention 1-3 bad things about the antibiotic handbook as it is now. Please write down reasons and suggestions for improvements:
a) (textbox)
b) I do not know
