# Supplementary figures and images for: Cross-sectional survey on the use and impact of the Danish national antibiotic use guidelines for companion animal practice
Source: Acta Vet Scand. 2017 Dec 11;59:81. doi: 10.1186/s13028-017-0350-8 (PMC5725655; doi:10.1186/s13028-017-0350-8)

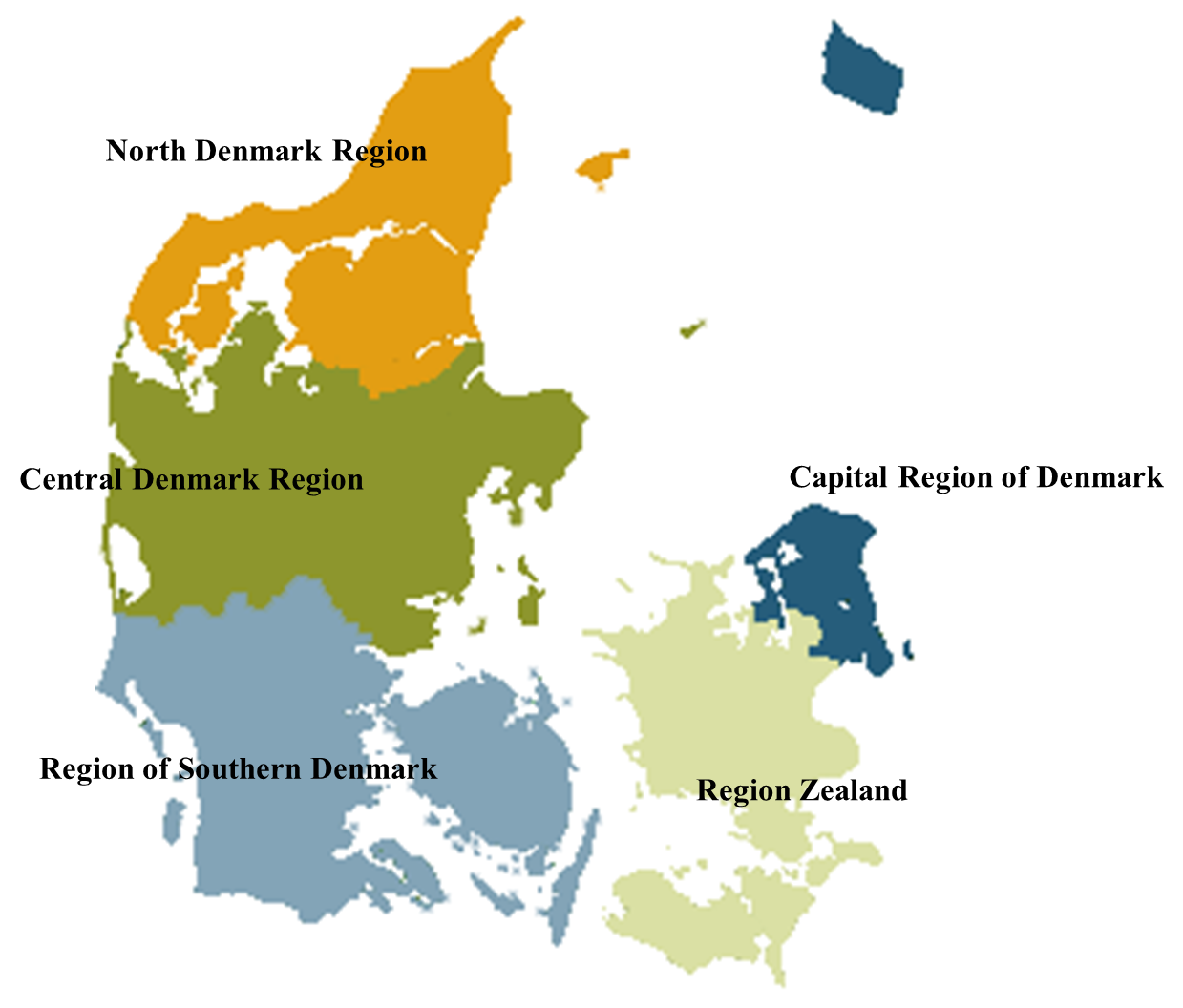

Supplement: Supplementary file 2 — Additional file 2. Map of the five Danish regions. [file 13028_2017_350_MOESM2_ESM.png]
